# Impact of KRAS Mutation on Survival Outcome of Patients With Metastatic Colorectal Cancer in Jordan

**DOI:** 10.7759/cureus.33736

**Published:** 2023-01-13

**Authors:** Mohammad S Alkader, Rashed Z Altaha, Sinan A Badwan, Anees I Halalmeh, Muna H Al-Khawaldeh, Mousa T Atmeh, Eslam H Jabali, Ola Attieh, Hana S Al-Soudi, Lean A Alkhatib, Mohammad T Alrawashdeh, Aseel F Abdelqader, Omar Y Ashokaibi, Ahmed A Shahin, Fadi M Maaita

**Affiliations:** 1 Department of Clinical Oncology, Jordanian Royal Medical Services, Amman, JOR; 2 Department of Internal Medicine, Jordanian Royal Medical Services, Amman, JOR; 3 Department of Nuclear Medicine, Jordanian Royal Medical Services, Amman, JOR; 4 Department of Pathology and Laboratory Medicine, Princess Iman Research and Laboratory Sciences Center, King Hussein Medical Center, Amman, JOR; 5 Department of Colorectal Surgery, Jordanian Royal Medical Services, Amman, JOR

**Keywords:** metastatic colorectal cancer, kras, colorectal cancer, impact of kras on prognosis, impact of kras on survival outcome, mutant kras, jordan, kaplan-meier survival curves, kras mutation, wild-type kras

## Abstract

Background

Colorectal cancer (CRC) is the most prevalent cancer in males, with an incidence rate (IR) of 13.1%, and the second most prevalent cancer in females, with an IR of 8.4%, coming after breast cancer in Jordan. The present study was motivated by conflicting clinical data regarding the prognostic impact of Kirsten rat sarcoma viral oncogene homolog (KRAS) mutation in patients with metastatic colorectal cancer (mCRC). Our study aimed to investigate if KRAS mutation conferred a negative prognostic value in Jordanian patients with mCRC.

Materials and methods

The current study is a retrospective study that collected data from a cohort of 135 mCRC patients diagnosed between 1 January 2017 and 1 January 2022 at our Oncology Department at the Jordanian Military Cancer Center (MCAC) using our patients' electronic medical records. The last follow-up date was 1 September 2022. From the cohort, we obtained data regarding age, sex, date of diagnosis, metastatic spread, KRAS status, either mutated KRAS or wild-type KRAS, and location of the primary tumor. All patients underwent tumor tissue biopsies to determine KRAS mutational status based on quantitative polymerase chain reaction and reverse hybridization from an accredited diagnostic laboratory at Jordan University Hospital. Statistical analysis was carried out to address the associations between* *KRAS mutation and the patients-tumor characteristics and their prognosis on survival.

Results

KRAS mutation was found in 40.3% of the participants in the study, and 56.7% had the wild type. There was a predilection of KRAS mutation, with 67% on the right side versus 33% on the left side (p* *= 0.018). Kaplan-Meier survival analysis showed worse survival outcomes in KRAS mutant patients (p* *= 0.002). The median overall survival in the KRAS mutant patients was 17 months (95% confidence interval (CI): 13.762-19.273) compared to 21 months (95% CI: 20.507-27.648) in patients with wild-type KRAS. Additionally, the Cox regression model identified that KRAS mutation carries a poorer prognosis on survival outcome hazard ratio (HR: 2.045, 95% CI: 1.291-3.237, p = 0.002). The test also showed statistical significance in the metastatic site (lung only). But this time, it was associated with a better survival outcome (HR: 0.383, 95% CI: 0.186-0.788, p = 0.009).

Conclusion

The present study shows that the presence of KRAS mutation has been found to negatively impact the prognosis and survival outcome of Jordanian patients with mCRC.

## Introduction

Colorectal cancer (CRC) is the most prevalent cancer in males, with an incidence rate (IR) of 13.1%, and the second most prevalent cancer in females, with an IR of 8.4%, coming after breast cancer in Jordan [1]. It is also the second most common cause of cancer-related mortality in males and females, with a mortality rate of 10.5% and 9%, respectively [1]. The overall five-year survival rate of metastatic colorectal cancer (mCRC) in the Jordanian population is 22.6% [2]. Considering the burden of CRC in Jordan's public health system in terms of prevalence and mortality, much effort has been made to address these problems by raising awareness campaigns of CRC's risk factors, signs, and symptoms and raising the importance of screening and early detection [3].

Kirsten rat sarcoma viral oncogene homolog (KRAS) is a member of the rat sarcoma viral oncogene (RAS) family. The other two isoforms of the RAS family are the Harvey rat sarcoma viral oncogene (HRAS) and neuroblastoma rat sarcoma viral oncogene (NRAS) [4]. KRAS is the best-known oncogene with the most frequent mutation rate among all cancers and is considered the most common oncogenic gene driver in human cancers, and it is most commonly seen in CRC, non-small cell lung cancer (NSCLC), and pancreatic cancer [5]. KRAS gene encodes a RAS GTPase protein called K-ras protein that plays a role in the epidermal growth factor receptor (EGFR) pathway. This sophisticated signaling pathway regulates cell growth, division, maturation, and death [4]. The natural, unchanged form is called wild-type KRAS, whereas the changed form is called mutant KRAS [6].

Constitutive-activated KRAS mutation results from a point mutation most commonly seen in codons 12 and 13 in CRC, increasing the risk of cancer development and causing cancer cells, if formed, to proliferate uncontrollably and metastasize in the body [7]. It is estimated that 30-50% of CRC patients harbor the mutated KRAS gene form [8]. These mutations are strongly associated with resistance to anti-EGFR therapy, such as cetuximab and panitumumab. It is prudent to determine the patient's KRAS status in CRC (i.e., wild type vs. mutant), as this will help to guide the treatment plan [9,10].

The mainstay treatment for mCRC is chemotherapy, targeted therapy, immunotherapy, and their combinations. It is well established that tailoring treatment to the molecular and pathologic features of the tumor improves overall survival (OS). Genomic profiling to detect somatic variants is essential because it identifies the type of therapy that may be effective [11].

## Materials and methods

Study design

This is a retrospective single-center study that included 135 mCRC patients diagnosed between 1 January 2017 and 1 January 2022 and followed up until 1 September 2022 at our Oncology Department at the Jordanian Military Cancer Center (MCAC) using our patients' electronic medical records. Our study aimed to investigate if KRAS mutation conferred a negative prognostic value in Jordanian patients with mCRC. The OS is the time between the date of metastasis and the date of death or the last follow-up date identified by the study. From the cohort, we obtained data regarding age, sex, date of diagnosis, metastatic spread, KRAS status, and location of the primary tumor.

The primary tumor location was identified based on the following: (1) right-sided: the primary tumor is located in the cecum, ascending colon, hepatic flexure, and transverse colon; (2) left-sided: the primary tumor is located in the splenic flexure, descending colon, sigmoid colon, and rectum.

The metastatic spread was categorized into liver only, lung only, liver and lung, peritoneum, > two organs involved (lung, liver, peritoneum), and others (including the brain, bone, non-regional lymph nodes, bladder, and reproductive organs). The date of diagnosis was the date of confirmed tissue biopsy and the presence of metastatic disease at first presentation. If the first presentation and initial diagnosis were other than metastatic, we included the date when metastasis first appeared as the date of diagnosis.

**Figure 1:**
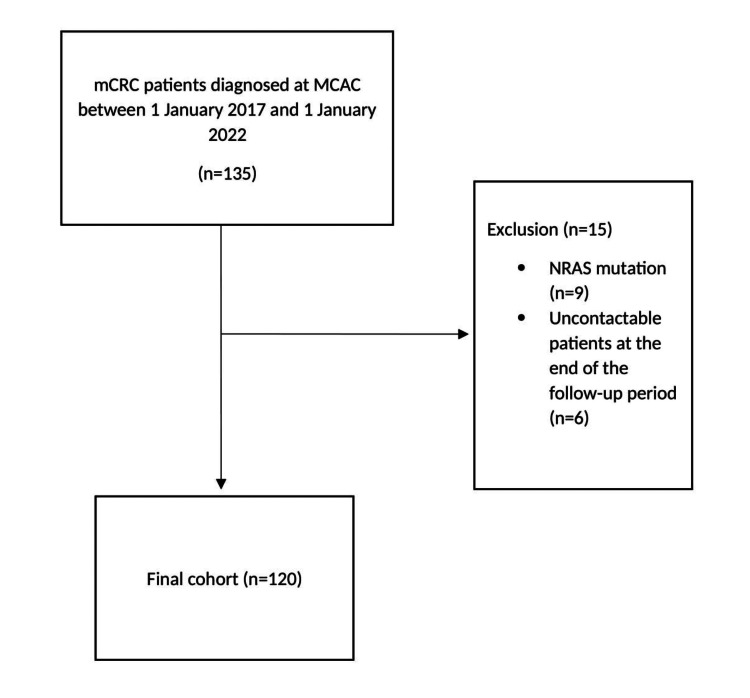
Flowchart of final patient selection mCRC: metastatic colorectal cancer; MCAC: Military Cancer Center; NRAS: neuroblastoma rat sarcoma viral oncogene; n: sample size.

Exclusion criteria were patients with NRAS mutation and uncontactable patients at the end of the follow-up period (Figure 1).

Ethics statement

This is an observational retrospective study. All patients were managed under routine clinical practice. The Institutional Review Board (IRB) at the Jordanian Royal Medical Services in Amman, Jordan approved the current study.

Tumor molecular analysis

All patients underwent tumor tissue biopsies to determine KRAS and NRAS mutation status based on quantitative polymerase chain reaction (PCR) and reverse hybridization from an accredited diagnostic laboratory at Jordan University Hospital. KRAS testing looks for 29 mutations in codons 12 and 13 of exon 2, codons 59, 60, and 61 of exon 3, and codons 117 and 146 of exon 4. NRAS testing looks for 22 mutations in codons 12 and 13 of exon 2, codons 59, 60, and 61 of exon 3, and codon 146 of exon 4.

Statistical analysis

Categorical data were expressed using frequencies and percentages. Numerical data were described as the mean and standard deviation or the median and range, as appropriate. The chi-squared test was used to assess the association between KRAS status and patients' disease characteristics.

Kaplan-Meier (KM) survival curves and log-rank assessment were used to compare the OS of mutant KRAS vs. wild-type KRAS. Cox regression analysis was conducted and stratified by patients' sex, age, primary tumor location, metastatic site, and KRAS status, and HR were calculated. All statistical analyses were analyzed using Statistical Package for the Social Sciences (SPSS) version 25.0 (IBM Corp., Armonk, NY). A p-value of <0.05 was considered statistically significant.

## Results

Descriptive statistics

**Figure 2:**
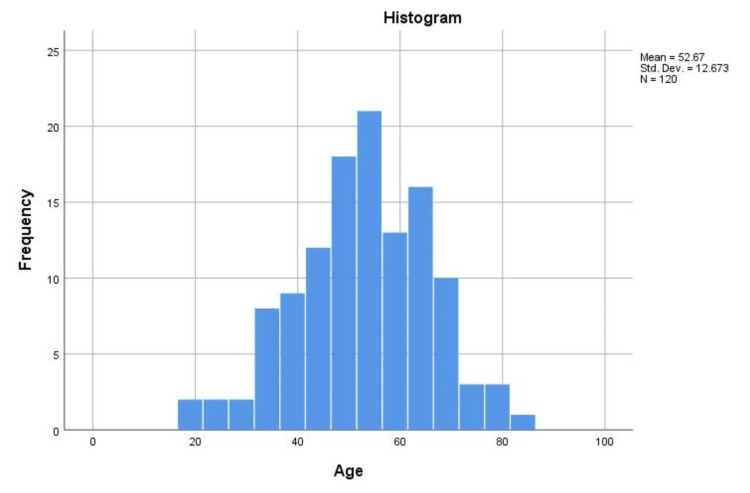
Age histogram N: total number of study participants.

Our final study participants included 120 patients with mCRC with a median age of 53.5 years (range = 19-83) (Table 1). The histogram distribution of age is shown in Figure 2.

**Table 1:** The mean and the median age of patients N: total number of study participants.

Age	
N	120
Mean	52.6
Median	53.5
Standard deviation	12.6
Minimum	19
Maximum	83

Baseline descriptive statistics of the 120 patients showed the following: (1) 60.8% and 39.1% were males and females, respectively; (2) 82.5% of the primary tumor location was on the left side, whereas 17.5% was found on the right side; (3) the metastatic sites were liver only (47.5%), lung only (18.3%), liver and lung (12.5%), > two organs involved (2.5%), other (5.8%), and peritoneum (13.3%); (4) KRAS status was mutant (43.3%) and wild type (56.7%); (5) there were nine total different codons associated with KRAS mutation, and the most common was gly12asp (36.5%), followed by gly12val (23.1%) (Table 2).

**Table 2:** Baseline descriptive statistics of patient-tumor characteristics KRAS: Kirsten rat sarcoma viral oncogene homolog; Gly: glycine; Asp: aspartate; Val: valine; Ser: serine; Lys: lysine; Asn: asparagine; Cys: cysteine; Ala: alanine; Thr: threonine; Glu: glutamate.

		Frequency	Percent
Status	Censored	24	20%
	Event	96	80%
Age groups	<40	19	15.8%
	40-60	67	55.8%
	>60	34	28.3%
Sex	Male	73	60.8%
	Female	47	39.2%
Site	Left	99	82.50%
	Right	21	17.50%
Metastatic spread	Liver only	57	47.50%
	Lung only	22	18.3%
	Liver & lung	15	12.50%
	> 2 organs involved	3	2.50%
	Other	7	5.8%
	Peritoneum	16	13.3%
KRAS status	Mutant	52	43.3%
	Wild-type	68	56.7%
KRAS codon	Gly12asp	19	36.5%
	Gly12val	12	23.1%
	Gly13asp	7	13.5%
	Gly12ser	4	7.7%
	Lys117asn	3	5.8%
	Gly12cys	3	5.8%
	Gly12ala	2	3.9%
	Ala59thr	1	1.9%
	Ala59glu	1	1.9%

Chi-square analysis between KRAS status and patient-tumor characteristics

Chi-squared test was used to assess the correlation between KRAS status and patient-tumor characteristics (age, sex, location of the primary tumor, and metastatic spread). KRAS mutation was found to have a predilection toward the right side (p = 0.018). There was no statistically significant difference in the distribution of age, sex, and metastatic spread according to KRAS status (Table 3).

**Table 3:** Chi-square analysis between KRAS status and patients-tumor characteristics KRAS: Kirsten rat sarcoma viral oncogene homolog.

KRAS status					
		KRAS mutant (n = 52) (%)	KRAS wild-type (n = 68) (%)	Total (n = 120)	P-value
Age in groups	<40	9 (47%)	10 (53%)	19	
	40-60	31 (46%)	36 (54%)	67	0.534
	>60	12 (35%)	22 (65%)	34	
Sex	Male	33 (45%)	40 (55%)	73	0.606
	Female	19 (40%)	28 (60%)	47	
Primary tumor location	Left	38 (38%)	61 (62%)	99	0.018
	Right	14 (67%)	7 (33%)	21	
	Liver only	25 (44%)	32 (56%)	57	
	Lung only	11 (50.0%)	11 (50.0%)	22	
Metastatic spread	Liver & lung	7 (47%)	8 (53%)	15	0.920
	>2 organs involved	1 (33%)	2 (67%)	3	
	Other	2 (29%)	5 (71%)	7	
	Peritoneum	6 (37.5%)	10 (62.5%)	16	

Survival analysis

**Figure 3:**
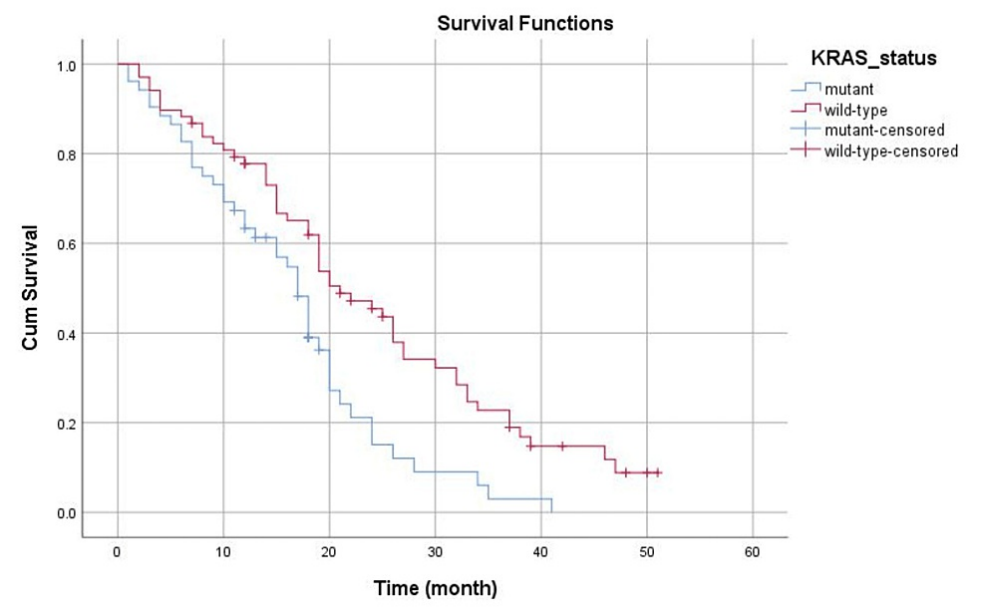
KM curve for overall survival according to KRAS status Red line: wild-type KRAS; blue line: mutant KRAS; Cum survival: cumulative survival; KM: Kaplan-Meier; KRAS: Kirsten rat sarcoma viral oncogene homolog.

At the end of the study period, the number of the surviving patients who had KRAS mutation was nine (17.3%), compared to 15 patients (22.1%) in wild-type KRAS. The median OS time was 17 months for mutant KRAS and 21 months for wild type (p = 0.002). The KM curve is shown in Figure 3.

**Figure 4:**
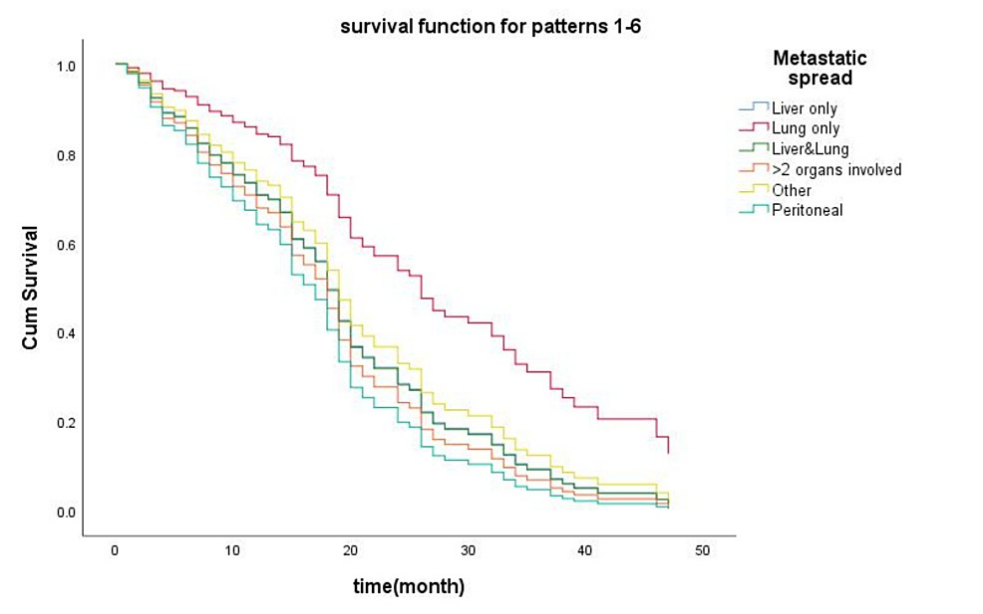
Survival curves for metastatic spread Red line: metastatic spread to lung only (paradoxically and the sole parameter in the present study that was associated with a better survival outcome; the other metastatic spread sites all had comparable results in terms of survival). Cum survival: cumulative survival.

We used the Cox proportional hazards regression method to investigate the effect of covariates such as age, sex, tumor location, metastatic spread, and KRAS mutation status on OS; we obtained an HR and 95% CI for each covariate (Table 4). Results showed that KRAS mutation carries a poorer prognosis on survival outcome (HR: 2.045, 95% CI: 1.291-3.237, p = 0.002). In addition, the test also showed statistical significance in the metastatic site (lung only). But this time, it was associated with a better survival outcome (HR: 0.383, 95% CI: 0.186-0.788, p = 0.009). The remaining covariates (age, sex, primary tumor location, and metastatic spread other than lung only) showed no statistical significance (all p > 0.05). KM survival curve for metastatic spread is shown in Figure 4.

**Table 4:** COX regression model KRAS: Kirsten rat sarcoma viral oncogene homolog.

	HR	95% CI		P-value
		Lower	Upper	
Sex (female)	0.832	0.542	1.275	0.398
KRAS (mutant)	2.045	1.291	3.237	0.002
Primary tumor location (right)	1.008	0.574	1.770	0.978
Liver only	0.776	0.427	1.412	0.407
Lung only	0.383	0.186	0.788	0.009
Liver and lung	0.782	0.354	1.726	0.542
>2 organs	0.876	0.234	3.282	0.844
Others	0.684	0.218	2.148	0.516
Age categorical (<40)	0.971	0.475	1.987	0.936
Age categorical (40-60)	1.030	0.625	1.699	0.907

## Discussion

The impact of KRAS mutation on the survival outcome of patients with mCRC has been a topic of much debate and research in oncology. Some studies have suggested that a KRAS mutation is associated with a poorer prognosis and reduced survival rates in these patients. For example, a meta-analysis by Levin-Sparenberg et al. published in 2020 that included data from 275 studies comprising 77,104 mCRC patients found that KRAS mutation had a negative prognostic value and was associated with significantly shorter overall survival in patients with mCRC [12].

Other studies have also demonstrated the negative impact of KRAS mutation on the survival outcome of patients with mCRC. A study published in 2020 by Dai et al. found that the KRAS mutation had a significantly worse OS rate than those without the mutation [13]. Another study published in 2020 by Ottaiano et al. found that KRAS mutation was an independent predictor of shorter OS in patients with mCRC [14].

Our study is in line with studies that showed a negative impact of KRAS mutation on survival outcomes in mCRC. Our analysis comprises data from 120 patients and represents one of the most extensive datasets available in Jordan. To the best of our knowledge, this is the first study in Jordan to analyze the impact of KRAS status on the survival outcomes of mCRC patients.

However, other studies have reported conflicting results with our study and have found no significant difference in survival outcomes between mutant and wild type in mCRC. For example, a study published in 2022 by Alghamdi et al. found that KRAS mutational status did not significantly affect OS or progression-free survival in patients with mCRC [15].

This inconsistency in the literature may be due to differences in study design, patient populations, and treatment regimens. It is also possible that KRAS mutation status may not be a predictor of survival outcomes on its own and may interact with other prognostic factors.

Interestingly, in our study, when looking for other predictors of survival outcomes, such as age, sex, location of the primary tumor, and metastatic spread, none of them showed a statistically significant difference in survival outcome, emphasizing the role of KRAS mutation as an independent predictor of shorter OS in patients with mCRC, findings similar to a study published by Ottaiano et al. [14].

Limitations

There were several limitations to the present study. It was a retrospective, single-center study. Microsatellite instability and other family members of the RAS that serve a function in CRC were excluded. The therapeutic regimens varied among the patients, which may have resulted in heterogeneity and affected the median OS survival, especially in patients with wild-type KRAS, as not all patients received monoclonal antibodies (mAbs) as first-line treatment (cetuximab and panitumumab) due to lack of availability.

## Conclusions

The present study shows that KRAS mutation has been found to negatively impact the prognosis and survival outcome of patients with mCRC in Jordan. The impact of KRAS mutation in mCRC will likely continue to evolve as research progresses. In particular, developing targeted therapies that specifically address the effects of KRAS mutations may hold promise for improving outcomes for these patients. Further research is needed to fully understand the mechanisms underlying the negative impact of KRAS mutation on survival in mCRC patients.
